# Assessing cognition and daily function in early dementia using the cognitive-functional composite: findings from the Catch-Cog study cohort

**DOI:** 10.1186/s13195-019-0500-5

**Published:** 2019-05-15

**Authors:** Roos J. Jutten, John E. Harrison, Philippe R. Lee Meeuw Kjoe, Silvia Ingala, R. Vreeswijk, R. A. J. van Deelen, Frank Jan de Jong, Esther M. Opmeer, André Aleman, Craig W. Ritchie, Philip Scheltens, Sietske A. M. Sikkes

**Affiliations:** 1 Alzheimer Center Amsterdam, Department of Neurology, Amsterdam Neuroscience, Amsterdam UMC, Vrije Universiteit Amsterdam, Amsterdam, The Netherlands; 2 Metis Cognition Ltd, Park House, Kilmington Common, Wiltshire, UK; 3 Institute of Psychiatry, Psychology & Neuroscience, King’s College London, London, UK; 4 Department of Radiology and Nuclear Medicine Amsterdam UMC, Vrije Universiteit Amsterdam, Amsterdam, The Netherlands; 5 Department of Geriatrics, Spaarne Gasthuis, Haarlem, The Netherlands; 6 Department of Neurology, Erasmus Medical Center, Rotterdam, The Netherlands; 7 Department of Biomedical Sciences, University Medical Center Groningen, University of Groningen, Groningen, The Netherlands; 8 Department of Health and Social Work, University of Applied Sciences Windesheim, Zwolle, The Netherlands; 9 Centre for Dementia Prevention, University of Edinburgh, Edinburgh, UK; 10 Department of Epidemiology & Biostatistics, Amsterdam UMC, Vrije Universiteit Amsterdam, Amsterdam, The Netherlands

**Keywords:** Alzheimer’s disease, Cognition, Composite, Daily function, Dementia, Construct validation, Instrumental activities of daily living, Mild cognitive impairment, Outcome measures

## Abstract

**Background:**

The cognitive-functional composite (CFC) was designed to improve the measurement of clinically relevant changes in predementia and early dementia stages. We have previously demonstrated its good test-retest reliability and feasibility of use. The current study aimed to evaluate several quality aspects of the CFC, including construct validity, clinical relevance, and suitability for the target population.

**Methods:**

Baseline data of the Capturing Changes in Cognition study was used: an international, prospective cohort study including participants with subjective cognitive decline (SCD), mild cognitive impairment (MCI), Alzheimer’s disease (AD) dementia, and dementia with Lewy bodies (DLB). The CFC comprises seven existing cognitive tests focusing on memory and executive functions (EF) and the informant-based Amsterdam Instrumental Activities of Daily Living Questionnaire (A-IADL-Q). Construct validity and clinical relevance were assessed by (1) confirmatory factor analyses (CFA) using all CFC subtests and (2) linear regression analyses relating the CFC score (independent) to reference measures of disease severity (dependent), correcting for age, sex, and education. To assess the suitability for the target population, we compared score distributions of the CFC to those of traditional tests (Alzheimer’s Disease Assessment Scale–Cognitive subscale, Alzheimer’s Disease Cooperative Study–Activities of Daily Living scale, and Clinical Dementia Rating scale).

**Results:**

A total of 184 participants were included (age 71.8 ± 8.4; 42% female; *n* = 14 SCD, *n* = 80 MCI, *n* = 78 AD, and *n* = 12 DLB). CFA showed that the hypothesized three-factor model (memory, EF, and IADL) had adequate fit (CFI = .931, RMSEA = .091, SRMR = .06). Moreover, worse CFC performance was associated with more cognitive decline as reported by the informant (*β* = .61, *p* < .001), poorer quality of life (*β* = .51*, p* < .001), higher caregiver burden (*β =* − .51, *p* < .001), more apathy (*β* = − .36, *p* < .001), and less cortical volume (*β* = .34, *p =* .02*)*. Whilst correlations between the CFC and traditional measures were moderate to strong (ranging from *−* .65 to .83, all *p* < .001), histograms showed floor and ceiling effects for the traditional tests as compared to the CFC.

**Conclusions:**

Our findings illustrate that the CFC has good construct validity, captures clinically relevant aspects of disease severity, and shows no range restrictions in scoring. It therefore provides a more useful outcome measure than traditional tests to evaluate cognition and function in MCI and mild AD.

**Electronic supplementary material:**

The online version of this article (10.1186/s13195-019-0500-5) contains supplementary material, which is available to authorized users.

## Background

Alzheimer’s disease (AD) is the leading cause of dementia worldwide and has been the target of clinical trials and intervention studies for many years [1]. In the past decade, the research field has shifted towards earlier clinical stages of dementia and to predementia stages such as mild cognitive impairment (MCI) [2]. Remarkably, the selection of cognitive and functional outcome measures to evaluate treatment effects has not been adapted to the shift in treatment target populations. Measures originally designed for mild to severe dementia, such as the Alzheimer’s Disease Assessment Scale–Cognitive subscale (ADAS-Cog [3]), are still widely used as primary endpoints in MCI and early AD dementia trials. Several studies have shown that those older, traditional measures are insensitive to change over time in MCI and mild dementia [4, 5], as they focus on cognitive domains and everyday activities that are unaffected in those disease stages [5–7]. This limits their clinical relevance in the predementia window and leads to range restrictions in scoring (i.e., floor and ceiling effects) [4, 8]. Hence, researchers and regulatory agencies have expressed the urgent need for a sensitive measure that is capable of detecting clinically relevant changes at early clinical stages of AD [9–13]. The same holds for dementia with Lewy bodies (DLB), the second most common cause of dementia, of which both pathology and clinical manifestations show considerable overlap with AD [14, 15].

The Capturing Changes in Cognition (Catch-Cog) project was initiated to fulfill the need for a sensitive, clinically relevant outcome measure for use in MCI and mild dementia. We designed a novel cognitive-functional composite (CFC) measure in our expert working group, basing our selections on previously published work and input from MCI and dementia patients and caregivers [16]. The resulting CFC consists of a short cognitive test battery focusing on memory and executive functioning (EF) [17], as these are the cognitive domains that have been shown to decline in predementia and early stages of dementia [13]. The rationale of specific cognitive tests included in this battery has been described in more detail elsewhere [16]. Briefly, the selection was based on empirical evidence on their sensitivity to change, as reflected by the absence of floor and ceiling effects in MCI and mild AD [17]. To amplify its clinical relevance, we augmented the cognitive test battery with a previously validated everyday functioning measure: the Amsterdam Instrumental Activities of Daily Living Questionnaire (A-IADL-Q) [18]. The A-IADL-Q assesses the problems in cognitively complex everyday activities such as cooking, managing finances, and using technological devices [19, 20]. In item response theory analyses, these activities were found to be the activities most sensitive to cognitive decline [21]. The A-IADL-Q was previously demonstrated to be sensitive to the decline in dementia, as well as able to capture difficulties in instrumental activities of daily living (IADL) functioning in MCI and subjective cognitive decline (SCD) [21, 22].

Combining these cognitive and functional measures into the CFC summarizes cognitive and everyday performance and may thereby provide a single clinically relevant score. Both the Food and Drug Association (FDA) and the European Medical Agency (EMA) encourage the use of such composite endpoints to evaluate novel drug therapies and interventions [11, 12]. These agencies also stipulate that composite measures should be (1) carefully designed, (2) validated in an independent prospective cohort study, and (3) “bear some relevance to existing tools for which historical experience exists” [12]. In the Catch-Cog study, we have sought to meet these criteria by performing an extensive construct validation of the CFC. In a previous report, we demonstrated that the CFC exhibits high test-retest reliability and good feasibility of use [23], which are pivotal prerequisites for a reliable and valid outcome measure [24]. Having demonstrated this, we embarked on a longitudinal construct validation in an independent prospective cohort across the clinical spectrum from SCD to mild dementia. The main aim of this study is to validate the CFC in MCI and mild AD dementia stages, but we will also explore whether the CFC could be of use in individuals with SCD and DLB.

In the current study, we performed a psychometric evaluation of the CFC using baseline data of the Catch-Cog study. We aimed to evaluate several quality aspects of the CFC, including the construct validity, clinical relevance, and suitability for the target population. Therefore, we investigated the CFC’s factor structure and compared the CFC score with reference measures of disease severity such as informant reports and global cortical atrophy [16]. We also examined CFC score distributions in direct comparison to currently used tests, including the ADAS-Cog [3], the Clinical Dementia Rating scale Sum of Boxes (CDR-SB) [25], the Alzheimer’s Disease Cooperative Study–Activities of Daily Living scale (ADCS-ADL) [26], and the Alzheimer’s Disease Composite Score (ADCOMS) procedure [27].

## Methods

### Study design and participants

In this cross-sectional study, we employed baseline data from the Catch-Cog study, which is an international, multicenter, prospective cohort study. Participants (*N* = 184) were recruited via the (1) Alzheimer Center Amsterdam, Amsterdam UMC, location VU University Medical Center, The Netherlands (*n* = 102); (2) Alzheimer Center Erasmus Medical Center (EMC, *n* = 14), Rotterdam, The Netherlands; (3) University Medical Center Groningen (UMCG, *n* = 39), The Netherlands; and (4) the Centre for Dementia Prevention, Edinburgh, Scotland (*n* = 29). We recruited participants who met the research criteria for SCD [28], the clinical criteria for MCI due to AD [2], probable AD dementia [29], or DLB dementia [15]. Other inclusion criteria were (1) Mini-Mental State Examination (MMSE) score ≥ 18, (2) age ≥ 50, and (3) availability of a study partner who was able and willing to participate. Exclusion criteria were (1) presence of any other neurological disorder, (2) presence of a major psychiatric disorder such as severe personality disorder or depression (Geriatric Depression Scale score ≥ 6 [30]), (3) current abuse of alcohol and/or drugs, and (4) simultaneously participating in a clinical trial.

Before inclusion, participants had undergone a standard diagnostic work-up in their study center, including at least medical history, neurological examination, and cognitive assessment. Structural brain imaging was available for a subset of the study cohort. Diagnoses were performed during a multidisciplinary consensus meeting, containing at least a neurologist or psychiatrist and with neuropsychology input. In the UMCG, SCD, and MCI, participants were also recruited via advertisements in local newspapers. After responding to this advertisement, eligible participants were screened by a neuropsychologist and neurologist to investigate whether they met the criteria for SCD or MCI [28].

The Medical-Ethical Committee of the VU University Medical Center approved the study for all Dutch centers. The South East Scotland Research Ethics Committee approved the study for the Scottish site. All participants and study partners provided written and oral informed consent.

### The cognitive-functional composite

#### Cognitive component

The cognitive test battery of the CFC included the three ADAS-Cog memory subscales Word Recognition, Word Recall and Orientation [3]; the Controlled Oral Word Association Test (COWAT); category fluency test (CFT); Digit Span Backward (DSB) and Digit Symbol Substitution Test (DSST) [31]. During the word recognition test, the participant is required to learn a list of 12 words and identify these words when mixed among 12 other distracter words (one point for each incorrect response, score range 0–12). During word recall, the participant is given three trials to learn a list of ten high-imagery nouns (total score entails the average number of words not recalled across the three trials, score range 0–10). The orientation subtest includes eight questions regarding the participant’s orientation to person, place, and time (one point for each incorrect response, score range 0–8). The COWAT assesses the participant’s phonemic fluency skills using the letters D-A-T in The Netherlands or F-A-S in English and a total time of 60 s per letter (one point for each correct non-repeated word). The CFT examines the participant’s semantic fluency by requiring them to generate as many exemplars of the category animals within 60 s (one point for each correct unique animal). The DSB requires the participant to reproduce sequences of digits of increasing length in the reversed order (score range 0–12). The DSST is a timed EF test during which participants have to substitute as many digits by unique geometric symbols within 90 s (one point for each correct substituted symbol).

#### Functional component

The functional component consisted of the short version of the A-IADL-Q [21]. The A-IADL-Q is a computerized, informant-based questionnaire covering a broad range of complex IADL [19]. The short version consists of 30 items covering household, administration, work, computer use, leisure time, appliances, and transport activities. For each item, difficulty in performance is rated on a 5-point Likert scale (ranging from “no difficulty in performing this task” to “no longer able to perform this task”). Scoring is based on item response theory, a paradigm linking item responses to an underlying latent trait [32]. This results in a latent trait score (*z*-score), reflecting one’s level of IADL functioning, with higher scores indicating better IADL functioning [21].

#### CFC scoring

To create CFC scores, the directionality of the three ADAS-Cog subtest scores were reversed, so that higher scores reflected better performance. Subsequently, all cognitive subtest scores were transformed into *z*-scores with total group mean and standard deviation (SD) as reference values. The cognitive composite was computed as a weighted *z*-score of all seven cognitive subtests, whereas the functional component score was the A-IADL-Q score. The overall CFC composite score was computed as a weighted *z*-score of the cognitive composite and A-IADL-Q, with higher scores indicating better performance.

### Reference measures

#### Traditional tests of cognition and function

Traditional tests to compare the CFC with included the MMSE, ADAS-Cog-13, ADCS-ADL, and CDR-SB. The MMSE is a global cognitive screening test, with a total score ranging from 0 to 30 and higher scores reflecting better performance [33]. The ADAS-Cog-13 yields a measure of cognitive performance by combining ratings of 13 subtests (e.g., word lists recognition and recall, constructional praxis, object and finger naming). Total scores range from 0 to 85, with higher scores indicating more severe impairment [3]. The ADCS-ADL assesses the functional abilities affected in mild-to-moderate AD. For 23 different basic and instrumental activities, the levels of performance and independency during the past 4 weeks were rated by the study partner. Total scores range from 0 (non-performance or need for extensive help) to 78 (independent performance) [26]. The CDR has been developed for the staging of dementia severity. The participant’s cognitive and functional performance is rated in 6 areas: memory, orientation, judgment and problem solving, community affairs, home and hobbies, and personal care. Each area is rated as 0 (healthy), 0.5 (questionable dementia), 1 (mild dementia), 2 (moderate dementia), or 3 (severe dementia). Adding the rating of all boxes results in a total CDR-SB score ranging from 0 to 18, with higher scores reflecting severe dementia [25, 34]. The ADCOMS is a recently designed, statistically derived composite scoring procedure, consisting of two MMSE items (“orientation to time” and “copy design”), 4 ADAS-Cog subtests (delayed word recall, orientation, word recognition, and word recall) and all 6 CDR-SB subscores. All items are differentially weighted yielding a score ranging from 0 to 1.27 with higher scores implying greater impairment [27].

#### Reference measures of disease severity

Informant reports of disease severity included the Cognitive Function Instrument study partner version (CFI-SP) [35], Quality of Life in Alzheimer’s Disease (QoL-AD) [36], the short version of Zarit Burden Inventory (ZBI-12) [37], and the Apathy Evaluation Scale (AES) [38]. The CFI-SP includes 14 items on a decline in day-to-day cognitive and functional abilities compared to 1 year ago. Response options include “yes” (0), “no” (1), or “maybe” (0.5), with total scores ranging from 0 to 14. The QoL-AD consists of 13 items, rated on a 4-point scale. Total scores range from 13 to 52, with higher scores reflecting better quality of life. The ZBI is one of the most commonly used instruments for assessing the aspects of caregiver burden [37]. Each item was rated on a 5-point scale, with total scores ranging from 0 to 60 and higher scores suggesting greater caregiver burden. The AES consists of 18 statements about the participant’s thoughts, feelings, and activity, which are rated on a 4-point scale. Total scores range from 0 to 72, with higher scores indicating more severe apathy.

Magnetic resonance (MR) images were acquired locally at each center in 3 T scanners. A minimum acceptable protocol was approved and then optimized at each site due to scanner differences (see Additional file 1). The images were checked for quality by an experienced rater. Volumetric measurements were processed on 3D T1-weighted (3DT1) images with Statistical Parametric Mapping 12 (SPM12) software (Wellcome Trust Centre for Neuroimaging, University College London, UK) running in MATLAB 2011a (MathWorks Inc., Natick, MA, USA). Prior to processing, the origin in each scan was manually set to the anterior commissure. Scans were segmented into gray matter (GM), white matter (WM), and cerebrospinal fluid (CSF). Total GM (i.e., the sum of all GM voxels) and total intracranial volume (TIV) (i.e., the sum of GM, WM, and CSF volumes) were derived from the segmented images in native space (units in liter). Cortical volume was defined as the total GM volume normalized for head size, divided by TIV.

### Procedures

Study visits took place at the hospital or the participant’s home, depending on the participant’s preference. A trained rater assessed the cognitive tests according to standardized instructions, starting with the MMSE and followed by the cognitive part of the CFC (word recognition, orientation, CFT, COWAT, DSST, DSB, word recall) and the remaining ADAS-Cog-13 tests. In the meantime, the study partner completed the A-IADL-Q, ZBI, and QoL-AD independently on an iPad. Subsequently, the participant completed the QoL-AD on the iPad with assistance from the rater. Finally, the rater completed the ADCS-ADL and CDR interview with the study partner. The total duration of a complete assessment was approximately 90 min. A shortened protocol was used in the SCD and DLB participants, as it was not our purpose to compare the CFC to the traditional tests that were not designed to assess the progression in these groups. Therefore, SCD and DLB participants only underwent the MMSE and cognitive battery of the CFC whilst their study partner completed the A-IADL-Q.

#### MRI procedures

MR scans acquired less than 6 months prior to the study visit were available for a subset of the study cohort. These included at least 3D T1- and T2-weighted imaging (T2) and 3D fluid-attenuated inversion recovery (FLAIR). Participants without a recent MRI scan available but who agreed to undergo a structural MRI scan were also scanned at 3 T with the same structural sequences which took about 30 min.

### Statistical analyses

Statistical analyses were performed using SPSS version 22.0 (IBM Corp., Armonk, NY) and R Studio (R Core Team, 2018). Statistical significance level was set at *p* value < .05, unless otherwise indicated. Demographic and clinical differences between the groups were investigated using chi-square tests, one-way analyses of variance (ANOVA) followed by Hochberg’s post hoc tests, and independent *t* tests for measures only available for the MCI and AD group.

#### Construct validity and clinical relevance

We performed confirmatory factor analyses (CFA) including all CFC subtests to investigate the CFC’s underlying factor structure. We evaluated a single-factor, two-factor (memory and EF), and three-factor (memory, EF, and IADL) model. In the two-factor model, the memory factor included the word recognition, orientation, and word recall tests and the EF factor included the CFT, COWAT, DSST, DSB, and A-IADL-Q. The three-factor model had a similar memory and EF factor, except that the A-IADL-Q was excluded from the EF factor and included a separate factor. We compared these models using chi-square tests and by evaluating their Comparative Fit Index (CFI), root mean squares of error approximation (RMSEA), and standardized root mean square residual (SRMR) indices, with CFI ≥ .90, RMSEA < .08, and SRMR < .08 considered as adequate fit [39]. We hypothesized that the three-factor model would fit, based on preparatory work on the cognitive component showing two underlying factors [40], and A-IADL-Q reflecting one underlying factor [19]. As a sensitivity analysis, all aforementioned CFA model evaluations were repeated in a restricted sample of MCI and mild AD participants, as this was the primary target population of the CFC.

Next, we investigated the differences in CFC scores across diagnostic groups using ANOVA followed by Hochberg’s post hoc tests, to examine whether scores would decrease from SCD to dementia. We assessed the association between the CFC variables and reference measures of disease severity, by performing linear regression analyses for each reference measure (CFI-SP, QoL-AD, ZBI-12, and AES, as dependent) and CFC score, age, sex and education as independents. To evaluate the added clinical value of the A-IADL-Q, we also investigated a second model including the cognitive component score and A-IADL-Q score as separate independents. The association between CFC score and gray matter volume was assessed with a linear regression analysis correcting for age, sex, years of education, and scanner type. We computed Pearson’s correlation coefficients to investigate the relation between CFC scores and traditional cognitive and functional measures.

#### Quality for the target population

As the CFC was initially designed for MCI and mild AD dementia, the comparison analyses with traditional measures and ADCOMS were performed using the MCI and AD groups only. Using this sample, we compared histograms of score distributions of the CFC, traditional tests, and ADCOMS to inspect range restrictions in scoring. To allow for appropriate comparisons between the CFC components and traditional tests, the histograms for the ADAS-Cog, ADCS-ADL, and CDR-SB score distributions were based on the standardized scores. Additionally, we reported original score ranges and distribution parameters (percentiles, skewness, and kurtosis) for all tests.

## Results

### Descriptive characteristics

The total sample (*N* = 184) had a mean age of 71.8 ± 8.4 years, 42% were female, and mean years of education was 13.6 ± 3.8. The majority had a diagnosis of MCI (*n* = 80) or AD dementia (*n* = 78). Table 1 presents the demographic and clinical characteristics separately for each diagnostic group. Groups differed in terms of age (*F* = 2.99, *p* = .033), but there were no significant differences regarding sex and education. MMSE scores were lower for dementia (AD, 24.0 ± 3.3; DLB, 24.8 ± 3.1) compared to MCI (26.7 ± 2.3) and SCD (29.3 ± 1.2). The AD group also performed worse on the ADAS-Cog (mean difference 6.3 points, *p* < .001), ADCS-ADL (mean difference 3.8 points, *p* = .01) and CDR-SB (mean difference 2.4 points, *p* < .001) when compared to the MCI group. Study partners of AD participants reported worse CFI-SP scores (mean difference 2 points, *p* < .001), lower quality of life scores (mean difference 2.2 points, *p* = .026), higher caregiver burden (mean difference 3.7 points, *p* = .004), and higher apathy levels (mean difference 3.4 points, *p =* .039) compared to study partners of MCI participants.

**Table 1:** Descriptive characteristics and test scores separately for each diagnostic group

	SCD (*n* = 14)	MCI (*n* = 80)	AD (*n* = 78)	DLB (*n* = 12)	*p* value	Post hoc between group comparisons*
Descriptives						
Age	68.1 (6.6)	73.6 (8.0)	71.3 (9.1)	68.3 (6.1)	.033	Non-significant
Female (%)	8 (57.1%)	29 (36.3%)	39 (50%)	2 (16.7%)	.060	N.A.
Education	15.2 (4.8)	13.7 (3.6)	13.2 (4.0)	13.6 (3.3)	.36	N.A.
MMSE	29.3 (1.2)	26.7 (2.3)	24.0 (3.3)	24.4 (2.9)	< .001	SCD > MCI > AD; SCD >DLB
CFC components						
Cognitive composite	.88 (.50)	.19 (.50)	− .31 (.61)	− .35 (.71)	< .001	SCD > MCI > AD, SCD > DLB, MCI > DLB
A-IADL-Q	.91 (.79)	.39 (.73)	− .48 (.84)	− .65 (.90)	< .001	SCD > AD; MCI < AD; SCD > DLB; MCI > DLB
CFC score	.89 (.57)	.29 (.51)	− .39 (.61)	− .51 (.75)	< .001	SCD > MCI > AD; SCD > DLB; MCI > DLB
Reference measures						
CFI-SP	N.A.	6.9 (3.2)	4.9 (2.8)	N.A.	< .001	N.A.
QoL-AD	N.A.	33.8 (5.3)	31.6 (5.2)	N.A.	.026	N.A.
ZBI-12	N.A.	10.7 (7.7)	14.4 (7.9)	N.A.	.004	N.A.
AES	N.A.	39.9 (10.5)	43.3 (10.0)	N.A.	.053	N.A.
Traditional tests						
ADAS-Cog	N.A.	22.2 (6.8)	28.5 (7.6)	N.A.	< .001	N.A.
ADCS-ADL	N.A.	67.5 (7.8)	63.7 (8.8)	N.A.	.010	N.A.
CDR-SB	N.A.	2.7 (1.9)	5.1 (2.2)	N.A.	< .001	N.A.

*Abbreviations*: *ADAS-Cog* Alzheimer’s Disease Assessment Scale–Cognitive subscale, *ADCS-ADL* Alzheimer’s Disease Cooperation Study–Activity of Daily Living, *AES* Apathy Evaluation Scale, *A-IADL-Q* Amsterdam IADL Questionnaire, *CDR-SB* Clinical Dementia Rating Sum of Boxes, *CFC* cognitive-functional composite, *CFI-SP* Cognitive Function Instrument study partner version, *MMSE* Mini-Mental State Examination, *QoL* Quality of life, *ZBI* Zarit Burden Inventory

*Based on Hochberg’s post hoc tests

### Construct validity and clinical relevance

**Fig. 1:**
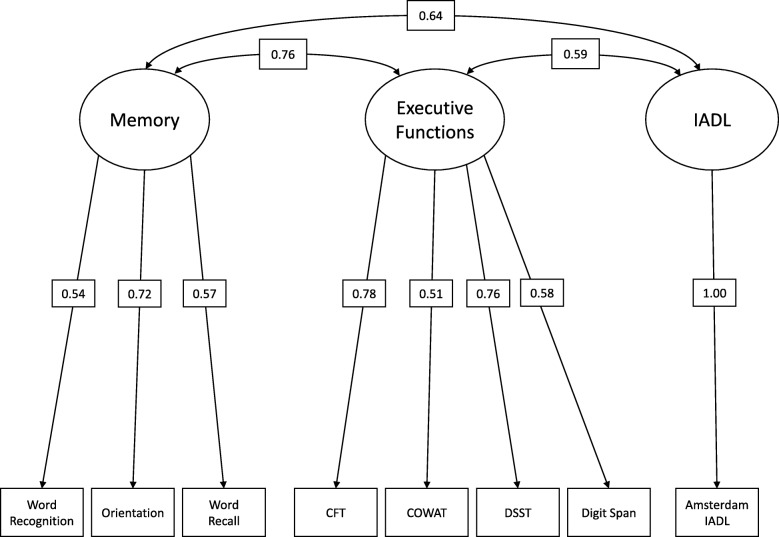
Path diagrams showing the three-factor structure of the CFC, including the covariance between domains and variables

CFA showed that the hypothesized three-factor model including memory, EF, and IADL had an adequate fit (CFI = .931, RMSEA = .091 (90% CI = .058–.124 and SRMR = .06), although the RMSEA index did not reach the predefined cutoff (< .08). The three-factor model (Fig. 1) described the data better than the single- or two-factor models (Table 2). Similar results were found after repeating the analyses in a restricted sample of MCI and mild AD participants (CFI = .918, RMSEA = .09 (90% CI = .053–.122 and SRMR = .06).

**Table 2:** Fit statistics for confirmatory factor analysis models

	Chi-square	df	*p* value	CFI	RMSEA [90%CI]	SRMR	Comparison with single-factor model	Comparison with two-factor model
Model 1: single factor	62.952	20	< .001	.891	.108 [.079–.139]	.070	N.A.	N.A.
Model 2: MEM + EF	52.543	19	< .001	.915	.098 [.067–.130]	.064	ChiSq(1) = 10.409, *p* = .001	N.A.
Model 3A: MEM + EF + IADL	45.269	18	< .001	.931	.091 [.058–.124]	.060	Chisq(2) = 17.683, *p < .*001	Chisq(1) = 15.873, *p <* .001

Model 1: Single factor based on all eight CFC subtests

Model 2: MEM = Word Recognition + Orientation + Word Recall, EF = Controlled Word Association Test + Category Fluency + Digit Symbol Substitution + Digit Span Backward + Amsterdam IADL Questionnaire

Model 3: MEM = Word Recognition + Orientation + Word Recall, EF = Controlled Word Association Test + Category Fluency + Digit Symbol Substitution + Digit Span Backward, IADL = Amsterdam IADL Questionnaire

*Abbreviations*: *CFI* Comparative Fit Index, *EF* executive functioning, *IADL* instrumental activities of daily living; *MEM* memory, *RMSEA* Root mean squares of error approximation, *SRMR* Standardized root mean square residual

**Fig. 2:**
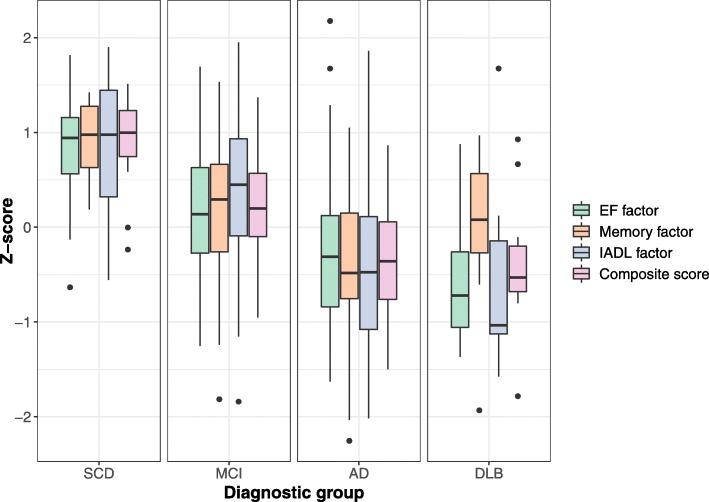
Box plots displaying scores on the CFC subcomponents (memory, EF, and IADL factor) and the overall CFC score, separately for each diagnostic group

As expected, overall CFC scores decreased concomitantly to progression across the spectrum from SCD (.89 ± .57) to MCI (.29 ± .51) and to AD or DLB dementia (AD, − .39 ± .61; DLB, − .52 ± .75), with significant differences between all groups except the two dementia groups (AD and DLB). A similar pattern was found for the cognitive composite score. A-IADL-Q scores were significantly lower for AD as compared to SCD and MCI, as well as for DLB compared to MCI and SCD (Table 1). Figure 2 visualizes the decreased scores across the diagnostic groups, with the cognitive composite divided into a memory and EF score according to the CFA results. It also shows that the CFC score is similar for the two dementia groups and that in AD, this score is driven by the memory factor rather than the EF factor, whereas the opposite is observed in DLB.

Lower CFC scores were associated with worse cognitive functioning as reported by the informant (corrected *β* = .61, *p* < .001), quality of life (corrected *β* = .51*, p* < .001), caregiver burden (corrected *β* = − .51, *p* < .001), and apathy level (corrected *β* = − .36, *p* < .001). Regression models including the cognitive composite and A-IADL-Q as separate scores demonstrated the added clinical value of the A-IADL-Q (Table 3). We found moderate-to-strong associations between the cognitive composite and ADAS-Cog (*r* = − .83; 95% CI = − .87 to − .77), the A-IADL-Q and ADCS-ADL (*r* = .65; 95% CI = .54–.73), and CFC score and CDR-SB (*r* = − .69; 95% CI = − .77 to − .56).

**Table 3:** Beta coefficients obtained from linear regression analyses relating CFC components to reference measures of disease severity

	CFI-SP				QoL-AD				ZBI-12				AES			
	Beta	95% CI		*p* value	Beta	95% CI		*p* value	Beta	95% CI		*p* value	Beta	95% CI		*p* value
Model 1																
CFC score	.61	.48	.74	< .001	.51	.37	.65	< .001	− .51	− .65	− .37	< .001	− .36	− .51	− .20	< .001
Age	.14	.02	.27	.028	− .06	− .20	.08	.388	− .08	− .21	.06	.280	− .01	− .16	.14	.913
Sex	.21	.08	.34	.002	.10	− .04	.24	.171	− .19	− .33	− .05	.009	− .19	− .34	− .04	.014
Education	− .04	− .17	.09	.578	− .13	− .27	.02	.087	.20	.06	.34	.006	.05	− .10	.21	.507
Model 2																
Cognitive composite	− .09	− .24	.06	.226	− .10	− .26	.07	.254	− .02	− .19	.15	.815	− .01	− .20	.18	.915
A-IADL-Q	.72	.58	.86	< .001	.62	.46	.77	< .001	− .52	− .68	− .36	< .001	− .37	− .55	− .19	< .001
Age	.11	− .01	.23	.066	− .09	− .22	.04	.182	− .06	− .19	.08	.409	.01	− .14	.16	.945
Sex	.18	.06	.30	.004	.07	− .06	.20	.305	− .17	− .31	− .03	.017	− .18	− .33	− .03	.021
Education	.04	− .09	.16	.552	− .06	− .20	.08	.405	.16	.02	.30	.030	.02	− .14	.18	.791

*Abbreviations*: *AES* Apathy Evaluation Scale, *A-IADL-Q* Amsterdam IADL Questionnaire, *CFC* cognitive-functional composite, *CFI*-SP Cognitive Function Instrument study partner version, *QoL* Quality of life, *ZBI* Zarit Burden Inventory

**Fig. 3:**
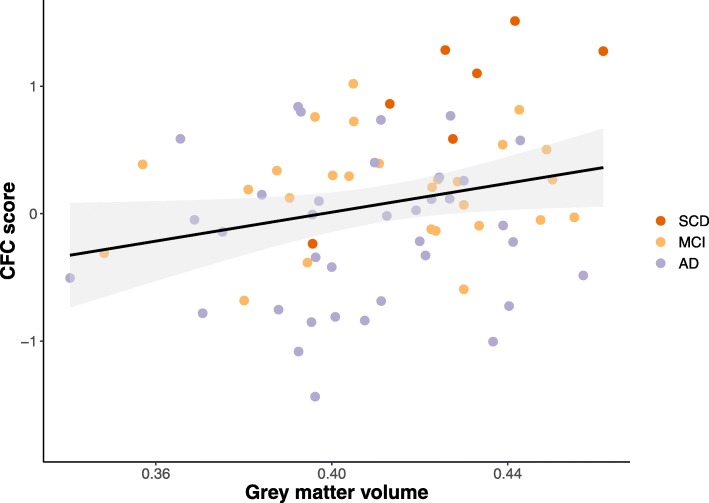
Scatterplot displaying the correlation between the CFC score and gray matter volume (corrected for total intracranial volume)

Brain MR images were available for 70 participants (*n* = 7 SCD, *n* = 27 MCI, and *n* = 36 AD). Linear regression analyses showed a significant association between normalized GM volume and CFC score (corrected *β =* .34, *p = .*01, Fig. 3), indicating that worse performance on the CFC was related to less cortical volume independently of age, sex, education, and scanner type.

### Quality for the target population

**Fig. 4:**
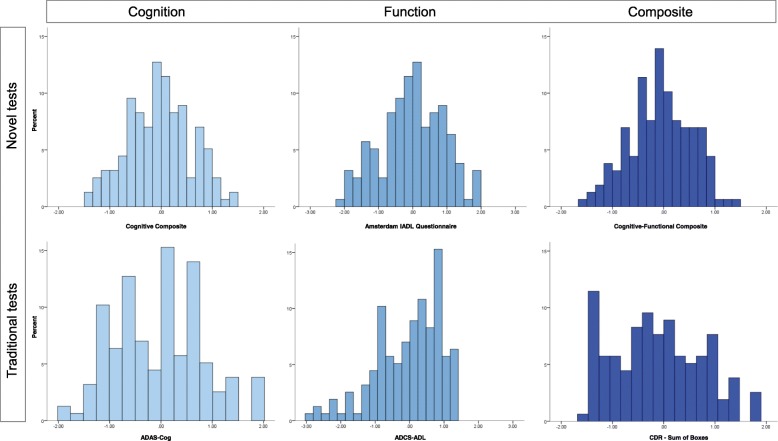
Score distributions of the CFC components and traditional tests in a combined sample of MCI and mild AD subjects (*n* = 158). Scores are standardized using the total sample mean and standard deviation as reference values

**Fig. 5:**
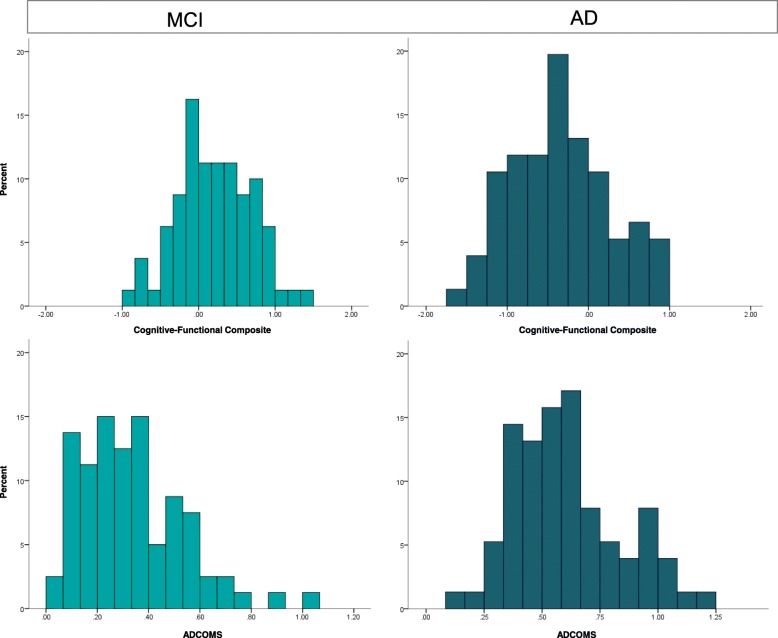
Score distributions of the CFC and ADCOMS, separately for MCI (*n* = 80) and mild AD dementia (*n* = 78)

Histograms including the total MCI and AD sample (*n* = 158) showed expected floor and ceiling effects in scoring for all the traditional measures (Fig. 4). Range restrictions were especially apparent for the ADAS-Cog and ADCS-ADL (Table 4). For example, for the ADCS-ADL, 35% of the participant scores were at the maximum end of the scale (between 70 and 78). By comparison, all CFC components showed normal distributions without range restrictions in scoring. Figure 5 displays a direct comparison between the ADCOMS and CFC score distributions, separately for the MCI and mild AD dementia group. Despite strong correlations between the ADCOMS and CFC scores (*r* = − .76; 95% CI = − .82 to − .68), it can be seen that the ADCOMS is more influenced by ceiling effects in scoring, particularly in the MCI subgroup.

**Table 4:** Score ranges, percentiles, and distribution parameters for the traditional test scores and CFC scores

	Score range		Percentiles							Distribution	
	Original	Current sample	5	10	25	50	75	90	95	Skewness	Kurtosis
ADAS-Cog	0–80	6–47	14	16	19	26	30	35	40	.236	.217
ADCS-ADL	0–78	39–78	47	55	60	68	73	75	77	− .902	.477
CDR-SB	0–18	0–12	.5	.7	2.0	3.5	5.5	7.0	8.7	.596	.142
ADCOMS	0–1.47	.05–1.21	.10	.15	.28	.43	.61	.86	.99	.620	− .042
A-IADL-Q	N.A.	− 2.01–1.95	− 1.71	− 1.32	− .64	.00	.63	1.04	1.42	− .091	− .457
Cognitive Composite	N.A.	− 1.44–1.33	− 1.13	− .88	− .52	− 0.04	.40	.81	.96	− .046	− .445
CFC	N.A.	− 1.51–1.37	− 1.14	− .87	− .42	− 0.04	.38	.78	.88	− .136	− .440

*Abbreviations*: *ADAS-Cog* Alzheimer’s Disease Assessment Scale–Cognitive subscale, *ADCOMS* Alzheimer’s Disease Composite Score, *ADCS-ADL* Alzheimer’s Disease Cooperation Study–Activity of Daily Living, *CDR-SB* Clinical Dementia Rating Sum of Boxes, *CFC* cognitive-functional composite

## Discussion

We performed a psychometric evaluation of the novel CFC. Factor analyses confirmed the underlying structure of the CFC, reflecting the domain memory, EF, and IADL. The associations that we found between the CFC score and reference measures of disease severity further supported the construct validity and clinical relevance of the CFC. We also demonstrated that the CFC scores yielded fewer range restrictions in scoring as compared to traditional tests of cognition and function, indicating a better quality for the target population.

Construct validity, clinical relevance, and suitability for the target population are important quality aspects for clinical outcome measures [41]. Construct validity refers to whether an instrument measures what it intends to measure [24]. As a first step to assess this, we performed a CFA which confirmed that our novel combination of tests can be described by a memory, EF, and IADL component. Interestingly, we observed that the two cognition factors contributed differentially to the overall CFC score as seen in AD versus DLB, with the AD group scoring worse on the memory component compared to the EF component and the DLB group vice versa. Notably, this is in line with the clinical pictures of both diseases with memory problems as the most prominent symptoms of early AD as opposed to more predominant EF problems in early DLB [14]. These results further confirm that the CFC score is an adequate reflection of the dimensionality of the construct to be measured.

Another aspect of construct validity is testing whether scores on an instrument are associated with scores on instruments that measure a similar construct [24]. We found moderate-to-strong associations with the traditional measures of cognition and function, which supports the construct validity of the CFC. It should, however, be noted that cognitive composite included three ADAS-Cog measures, which probably accounts for the strong association we found between these measures. We also demonstrated that the CFC is associated with other clinically relevant measures of disease severity, such as cognitive decline as reported by the informant, quality of life, caregiver burden, and apathy. Additionally, the association between CFC score and GM volume, which has shown to be a good biomarker of neurodegeneration in AD [42, 43], illustrates that the CFC assesses a construct that is related to the underlying disease process. Taken together, these associations suggest that several clinically relevant aspects of the disease and its severity are captured by the CFC.

Quality for the target population was evaluated by inspecting range restrictions in scoring in our MCI and early dementia sample. This population largely corresponds with “stage 3 patients” as described in the recently proposed NIA-AA clinical staging scheme and aligned FDA guidance [11, 44]. We found that both the traditional tests and the recently designed ADCOMS showed ceiling effects in scoring in this stage, indicating that these participants showed a high level of functioning. These ceiling effects hamper the measurement of changes and especially the measurement of potential improvement due to treatment. In contrast, the score distributions of the CFC component were normally distributed, showing potential to indicate both decline and improvement with respect to baseline measurements. Therefore, these cross-sectional results support that the CFC is a promising measure with which to assess changes over time without exhibiting the limitations of traditionally used tests.

Several other endeavors have been undertaken to design and validate composite measures that are more appropriate to assess changes earlier clinical stages of AD. These composites range from purely statistically driven [27, 45] to more theoretically based such as the preclinical Alzheimer’s cognitive composite (PACC) [46] and the Alzheimer prevention initiative cognitive composite (APCC) [47]. The PACC and APCC focus on preclinical stages of AD and do not include a measure of daily function. However, measuring everyday functioning is highly relevant in the MCI and dementia stages, as evolving IADL problems are an important clinical hallmark in the transitional stage from MCI to dementia and predict a future decline in dementia [7]. The ADCOMS procedure includes a functional measure (CDR-SB), and the fact that previously changes were detected in a clinical trial [27] supports the idea that adding a functional measure advances a cognitive outcome measure. However, the ADCOMS selection has been largely driven by statistical considerations rather than its content, and therefore, its clinical relevance is as yet uncertain. It is our view that the CFC can contribute to the existing initiatives to improve cognitive measurement in the MCI and mild dementia stages, as its composition has been based on both theoretical constructs (i.e., the combination of cognition and IADL measure) and empirical research [17, 22].

There are some limitations to consider. First, not all MCI and AD participants had AD biomarkers available, and therefore, it is unknown whether they had AD pathology. However, in these circumstances, we relied on an extensive clinical assessment and excluded participants with other conditions that could have caused or contributed to the cognitive or functional symptoms. Second, some heterogeneity in our sample may have been caused by minor differences in the recruitment strategies employed across the centers as well as from including SCD and DLB participants. Consequently, our sample may not perfectly mirror the composition of an ideal clinical trial sample. It should also be noted that the SCD and DLB samples were relatively small and that the CFC investigations in these groups were of explorative nature. This limits the interpretation of the CFC results in those groups. Additionally, the sample size of participants with an MRI scan available was relatively small, as this was not required for participation in our study. Therefore, we should be careful with interpreting these findings, particularly in the SCD group. A further limitation is that we have only investigated a single weighting method to create the CFC score, whereas it is likely that the optimal scoring method involves different weights for the different components. For example, our data showed that the memory component seems to be relatively easy compared to the EF component, which might be something that we need to account for when tracking the changes over time, in particular for the MCI group. Lastly, the fact that the CFC in its current composition focuses less on other domains than episodic memory and EF may limit its usefulness for measuring progression at more severe stages of dementia.

Strengths of this study include our construct validity approach, which is a unique aspect of the Catch-Cog study. Given the lack of a gold standard for “disease severity,” we used different reference measures of disease severity to compare the CFC with, which led to converging evidence for the clinical relevance of the CFC. Additionally, we were able to perform a direct head-to-head comparison between the CFC and traditional tests, which, to our knowledge, has never been done in previous studies. The advantage of this is to reveal both the strengths and weaknesses of different clinical measurements. Furthermore, our investigation of the CFC in an independent, prospective cohort is an essential aspect of this study. Although the CFC consists of tests that have been validated as part of other test batteries and across several study cohorts, it cannot be assumed that all measures perform similarly in a novel composition. For example, tests may perform differently when assessed in a different test order. An independent validation of a novel composite measure such as the CFC measure is thus needed to enhance future implementation. We are currently assessing the CFC longitudinally in our Catch-Cog prospective study cohort, and we will investigate its sensitivity to change after 3, 6, and 12 months and compare its sensitivity with that of the traditional tests. This longitudinal data will also enable us to explore whether different weights for the subtests can improve the sensitivity to change over time, as well as whether different weights are useful when tracking a change in different diagnostic groups. For example, putting more weight on the cognitive parts and activities of daily living that decline relatively late in the disease course may enhance the use of the CFC to track progression in later disease stages.

## Conclusions

We demonstrated that the CFC has good construct validity and captures clinically relevant aspects of disease progression. We also showed its improved suitability for the target population as compared to traditional tests, as reflected by fewer range restrictions in scoring. These findings illustrate that the CFC has good potential to be a sensitive, clinically meaningful outcome measure. It is therefore better indicated for use to evaluate cognition and function as compared to traditional tests, as in line with the recent FDA recommendations. Ultimately, the CFC can yield a more accurate and useful measurement of clinically relevant changes, which will aid the monitoring of disease progression and evaluation of novel treatments.

## Additional file

Additional file 1: MRI settings. (DOCX 13 kb)

## Abbreviations

AD: Alzheimer’s disease
ADAS-Cog: Alzheimer’s Disease Assessment Scale–Cognitive subscale
ADCOMS: Alzheimer’s Disease Composite Score
ADCS-ADL: Alzheimer’s Disease Cooperative Study–Activities of Daily Living
AES: Apathy Evaluation Scale
A-IADL-Q: Amsterdam IADL Questionnaire
Catch-Cog: Capturing Changes in Cognition
CC: Cognitive composite
CDR-SB: Clinical Dementia Rating scale Sum of Boxes
CFA: Confirmatory factor analysis
CFC: Cognitive-functional composite
CFI-SP: Cognitive Function Instrument - study partner version
CFI: Comparative Fit Index
CFT: Category fluency test
COWAT: Controlled Oral Word Association Test
CSF: Cerebrospinal fluid
DLB: Dementia with Lewy bodies
DSST: Digit symbol substitution test
EF: Executive functioning
EM: Episodic memory
FLAIR: Fluid-attenuated inversion recovery
GM: Gray matter
IADL: Instrumental activities of daily living
IRT: Item response theory
MCI: Mild cognitive impairment
MMSE: Mini-Mental State Examination
MRI: Magnetic resonance imaging
QoL: Quality of life
RMSEA: Root mean squares of error approximation
SCD: Subjective cognitive decline
SRMR: Standardized root mean square residual
TIV: Total intracranial volume
WM: White matter
WM: Working memory
ZBI: Zarit Burden Inventory
