# Supplementary material for: Assessing cognition and daily function in early dementia using the cognitive-functional composite: findings from the Catch-Cog study cohort
Source: Alzheimers Res Ther. 2019 May 15;11:45. doi: 10.1186/s13195-019-0500-5 (PMC6521452; doi:10.1186/s13195-019-0500-5)
Supplement: Supplementary file 1 — MRI settings. (DOCX 13 kb) [file 13195_2019_500_MOESM1_ESM.docx]

**Additional File 1: MRI settings per scanner type**

| MRI sequence | Settings | | |
| --- | --- | --- | --- |
|  | Philips 3T | GE 3T | Siemens 3T |
| 3DT1 | Sagittal 3D turbo field echo sequence (1.00 mm isotropic voxels, TR = 7.9 ms, TE = 4.5 ms, FA = 8 degrees) | Sagittal 3D FSPGR (1.00 mm isotropic voxels, TR = 8.2 ms, TE = 3.2 ms / TI = 450 ms, FA = 12 degrees) | 3D MPRAGE (1.00 mm x 1.00 mm x 1.00 mm voxels, TR = 2300 ms, TE = 3 ms) |
| 3D Fluid-attenuated inversion recovery | 3D sagittal VISTA sequence (1.12 mm isotropic voxels, TR = 4800 ms, TE = 279 ms, inversion time = 1650 ms) | 3D sagittal CUBE sequence (1.2 mm isotropic voxels, TR = 8000 ms, TE = 130 ms, inversion time = 2340 ms) | 3D sagittal SPACE sequence (1.2 mm isptropic voxels, TR = 5000 ms, TE = 387 ms ms, inversion time = 2340 ms) |
| 2D PD-T2: | Oblique axial orientation, 45-48 slices of 3mm thickness (TR = 3925 ms, TE = 19 and 100 ms) | Oblique axial orientation, 45-48 slices of 3mm thickness (TR = 8600 ms, TE = 20 and 112 ms) | Oblique axial orientation, 45-48 slices of 3mm thickness (TR = 4730 ms, TE = 20 ms) |

*TR: repetition time; TE: echo time; FA: flip angle.*
